# Does information disclosure among public hospitals stimulate medical cost change efforts? A pilot study in Shanghai

**DOI:** 10.1186/s12913-023-09510-8

**Published:** 2023-05-24

**Authors:** Ya-jing Chen, Rui-Xin Wang, Jin-yan Tang, Xiao-hua Ying

**Affiliations:** 1 School of Public Health, Fudan University, 130 Dongan Road, Shanghai, China; 2 Key Laboratory of Health Technology Assessment, Ministry of Health, Fudan University, 130 Dongan Road, Shanghai, China

**Keywords:** Information, Disclosure, Interhospital, Medical costs

## Abstract

**Background:**

In 2013, the Shanghai Hospital Development Center issued a policy to advocate public hospitals to report their information about costs on diseases. The objective was to evaluate the impact of interhospital disclosure of costs on diseases on medical costs and compare costs per case following information disclosure between hospitals of different rankings.

**Methods:**

The study uses the hospital-level performance report issued by Shanghai Hospital Development Center in the fourth quarter of 2013, which covers quarterly aggregated hospital-level discharge data from 14 tertiary public hospitals participating in thyroid malignant tumors and colorectal malignant tumors information disclosure from the first quarter of 2012 to the third quarter of 2020. An interrupted time series model with segmented regression analysis is employed to examine changes in quarterly trends with respect to costs per case and length of stay before and after information disclosure. We identified high- and low-cost hospitals by ranking them on a costs per case basis per disease group.

**Results:**

This research identified significant differences in cost changes for thyroid malignant tumors and colorectal malignant tumors between hospitals after disclosing information. A hospital’s discharge costs per case for thyroid malignant tumors increased significantly among top-cost hospitals (1629.251 RMB, P = 0.019), while decreased for thyroid and colorectal malignant tumors among low-cost hospitals (-1504.189 RMB, P = 0.003; -6511.650 RMB, P = 0.024, respectively).

**Conclusion:**

Our findings indicate that information disclosure of costs on diseases results in changes in discharge costs per case. And low-cost hospitals continued to maintain their leading edge, whereas the high-cost hospitals changed their position in the industry by reducing discharge costs per case after information disclosure.

**Supplementary Information:**

The online version contains supplementary material available at 10.1186/s12913-023-09510-8.

## Introduction

In China, public hospitals, which provide over 80% of overall inpatient and outpatient services, play the most important role in service delivery [1]. However, once the profit-seeking mechanism of the public hospital was established, the motivation of profit-seeking became pervasive among public medical service providers, leading to a significant increase in public hospital revenue and bringing in substantial negative impact by increased treatment doses and frequency, especially for drugs and services [2]. When the information transfer between hospitals is lack, there is a greater chance of hidden malpractices or problems with a hospital’s operation. Under the settings of information asymmetry, one of the key tools to strengthen the supervision and governance of the medical service market, which is advocated by both scholars and practitioners, is information disclosure among hospitals [3–5].

Under the fee-for-service payment system, hospitals have the incentive to increase the volume of services and to choose treatments with a greater profit margin [6]. The Shanghai Hospital Development Center is responsible for the investment, construction, operation, management and assessment of municipal public hospitals. To promote the development of hospitals, the center regularly evaluate the performance of hospitals and give them feedback on the assessment results, including the performance of the hospital managers and overall operation of the hospitals, the performance of service delivery for typical diseases, and performance of hospital internal management system. Since 2013, the Shanghai Hospital Development Center has been collecting costs on diseases information of all public hospitals and reported the results back to the participating hospitals. Indeed, those whose data are being reported is what information receivers, including hospital managers, directors, and competitors. Such reporting is based on the assumption that giving providers more information will enable them to make better decisions. The theory is that providers will make a trade-off between revenue maximization and reputation.

The rationale behind information disclosure is compelling [7]. Interhospital information disclosure has the function of signal transmission, which is to provide transparency, and to drive a direct provider response [8]. On the one hand, the cost information included in reports is important to hospital managers, because such data, including discharge costs per case, outpatient and emergency costs per case, and drug proportion, are directly relevant to the performance of Chinese public hospital directors [9]. One the other hand, on learning that they are labeled “low cost”, providers will respond to increase their profits owing to concerns about reputation [10, 11]. Research indicates that some organization whose performance is shown in a positive light by the reports, use the information for benchmarking and for internal monitoring of performance [12]. Hibbard et al. [13] focus on the correlation between public hospital performance information disclosure and reputation. They reported that making performance information public affects hospitals’ image and stimulates long-term improvements. In addition, we need to include culture as a factor, as there may be fundamental differences in the perceptions of privacy and expectations for information publicity. In China and other East Asian Countries, culture was the most important factor associated with preferences for information disclosure, and providers with superior abilities tended to voluntarily disclose information and change behavior for reputational reasons [14]. Furthermore, we make our hypothesis that the information exposed to an individual may have spillover impacts on his/her behavior. Based on the findings reported in the literature, we aim to test whether the interhospital disclosure of costs information impacts the behavior of a hospital. It is possible that hospitals operating with institutional similarities will adopt homogeneous behaviors [15].

In addition, a few studies have been conducted to evaluate the impacts of information disclosure on healthcare providers and patients and these studies reported conflicting results [16–18]. For example, with regard to nationwide evaluation of information disclosure, Schneider et al. [19] conducted qualitative research across the United State and found that publicly disclosed information on quality has improved the processes and outcomes in healthcare in limited ways in some settings, but these efforts have not led to “consumer choice” market prediction. Hibbard et al. [20] indicate that the disclosure of performance information appears to stimulate quality improvement measures in areas where performance is reported to be low. A review conducted by Marshall et al. [17] argued that disclosure of information on performance can be advocated as a mechanism to regulate providers of care, ensure accountability, encourage cost control, and promote quality improvement. Besides, in a study conducted in Taiwan [21], it was observed that the public reporting of hospital financial information led to less information asymmetry among insurers, thereby facilitating more reasonable allocation of total medical expenses based on the hospitals’ overview of their operations. Previous research models have found quality as a cost driver and study it in a context where hospitals are either sensitive to their own reputation or in a spatial competition framework. More recently, most studies have focused on the disclosure of cost information to a consumer audience with the expectation that consumers will use that information to choose their providers. However, few studies have examined the possibility of hospital behavior change in the wake of interhospital information disclosure. Further, the most effective form of information disclosure among hospitals remains to be determined through further evaluation.

Therefore, the principal objective of this study was to evaluate the impact of interhospital disclosure of costs on diseases based on hospital-level performance reporting on medical costs and compare costs per case following information disclosure between hospitals of different rankings. Additionally, in order to further explore the potential reasons for the change of costs, we also analyzed the drug costs and consumable costs. With the results of our study, we aim to provide an insight into the role of performance reporting in the healthcare industry.

## Materials and methods

### Study setting and design

This study uses interrupted time series with segmented regression analysis to evaluate the impact of information disclosure through hospital-level performance reporting. The intervention under study is the implementation of an internal policy in January 2013 that was passed at the Shanghai Hospital Development Center advocating hospitals to report information about costs on diseases. The Shanghai Hospital Development Center, as a municipal government bureau, is responsible for the investment, construction, operation, management and assessment of municipal public hospitals. In October 2013, the Shanghai Hospital Development Center released the first report to hospitals, covering medical cost information from January to August. From the fourth quarter of 2013, Shanghai Hospital Development Center began to publish quarterly reports for interhospital use. Hence, in this quarterly data-based study, the pre-intervention period included the first quarter of 2012 to the third quarter of 2013, whereas the post-intervention period included the fourth quarter of 2013 to the third quarter of 2020. This change was applied across 33 tertiary public hospitals in Shanghai with the exclusion of one hospital with missing data and four newly built hospitals in the suburban area.

### Data sources and measurements

Our data source included hospital-level costs from the first quarter of 2012 to the third quarter of 2020, collected from the hospital-level performance report issued by Shanghai Hospital Development Center, which was established in 2005. Before the disclosure of costs on diseases information of public hospitals in the fourth quarter of 2013, the report only included quarterly data at the hospital level, but did not include data for each disease. Therefore, we assume that before the disclosure of costs on diseases, the trend of expenses of each disease is consistent with the trend of total costs in each hospital. Accordingly, we considered the cost of each disease from the fourth quarter of 2013, based on the quarterly rate of change in the total costs, and estimated the costs per case for each disease before the disclosure of costs on diseases. Municipal-level public hospitals are established by the municipal government unit and are usually larger hospitals. The contents disclosed in the hospital-level performance report include the following information: the hospital name, medical costs (e.g., discharge costs per case, drug costs per case, and consumable costs per case), admission and discharge-related information per case, disease-related information (e.g., number of cases, and length of stay), and hospital characteristics (e.g., number of employees and number of beds) (supplementary Table 1).

During the study period, the diseases for which information was disclosed changed every year. The chosen disease took into account factors such as higher cost ratio, time required to disclose information, and no missing data among others. Accordingly, we selected thyroid malignant tumors and colorectal malignant tumors to study the effects of disease information disclosure on costs per case (supplementary Fig. 1).

We used discharge costs per case, drug costs per case, and consumable costs per case as the primary outcome variables and length of stay as secondary outcome. Analysis for the present study included hospitals participating in thyroid malignant tumors and colorectal malignant tumors information disclosure between 2012 and 2020 (14 tertiary public hospitals, of which 12 hospitals reported information on two diseases separately, and 10 hospitals reported on both diseases) for analysis. Subsequently, we separately analyzed the impact of information disclosure on discharge costs per case, drug costs per case, consumable costs per case, and length of stay across two diseases, as well as compared discharge costs per case, drug costs per case, consumable costs per case, and length of stay separately for each disease following information disclosure between hospitals of different rankings for the same disease. Hospitals were stratified according to quartiles of average costs per case in the fourth quarter of 2013, where Q1 indicated hospitals with the lowest costs and Q4 indicated hospitals with the highest costs [22] (Table 1).

**Table 1:** Descriptive statistics on sample hospitals and inpatients before and after the information disclosure

	Total (N = 14 hospitals)		Thyroid malignant tumors (N = 12 hospitals)			Colorectal malignant tumors (N = 12 hospitals)		
			Before	After		Before	After	
**Hospital characteristics**								
Hospital type, No. (%)								
General	13 (92.86)		11 (91.67)	11 (91.67)		11 (91.67)	11 (91.67)	
Specialty	1 (7.14)		1 (8.33)	1 (8.33)		1 (8.33)	1 (8.33)	
No. of beds, mean (SD)	1,630 (324.78)		1,426 (346.83)	1,665 (297.98)		1,473 (358.99)	1,683(351.45)	
No. of employees, mean (SD)	3,139 (778.90)		2,815 (636.91)	3,300 (786.56)		2,870 (690.26)	3,200 (834.40)	
Annual discharges per hospital, mean (SD)	88,964(23916.28)		66,953(18613.51)	97,255(25698.80)		67,553 (20348.88)	95,329 (28035.22)	
Annual costs per hospital [RMB, million], mean (SD)	3366.73 (1095.90)		2369.51 (596.22)	3833.67 (1203.73)		2356.53 (670.24)	3688.83 (1288.02)	
Length of stay [days], mean (SD)	7.23 (0.60)		8.62 (1.48)	6.82 (0.61)		8.66 (1.48)	6.89 (0.68)	
**Inpatient characteristics**								
Cases with targeted diseases, No.	218,928		14,246	106,517		13,241	84,924	
Proportion of cases with targeted diseases [‰], mean (SD)			11.47 (16.53)	13.38 (15.18)		9.87 (7.18)	10.57 (7.22)	
Costs per case with targeted diseases [RMB], mean (SD)			15144.22 (2957.82)	17927.70 (3000.38)		49761.75(10052.56)	57772.37 (13076.74)	
Proportion of costs on targeted diseases [‰], mean (SD)			3.62 (5.59)	6.94 (7.59)		12.11 (5.99)	14.30 (7.70)	
Drug costs per case with targeted diseases [RMB], mean (SD)			3464.79 (1659.31)	3022.68 (1283.89)		18313.48 (6880.50)	15637.21 (7036.72)	
Consumable costs per case with targeted diseases [RMB], mean (SD)			1692.70 (996.64)	2833.43 (1195.23)		15774.99 (3579.57)	18740.66 (7328.66)	
Length of stay per case with targeted diseases [days], mean (SD)			6.99 (1.79)	5.78 (1.53)		20.57 (3.62)	16.64 (4.29)	
**Subgroup characteristics by quartiles**								
Costs per case with targeted diseases [RMB], mean (SD)								
Q1 subgroup of hospitals (N = 3)			11791.28 (982.53)	16746.42 (2363.21)		40886.50 (5792.59)	48662.94 (8830.17)	
Q2 subgroup of hospitals (N = 3)			14005.46 (1432.52)	16756.35 (2707.67)		44275.03 (8009.01)	49967.12 (5722.54)	
Q3 subgroup of hospitals (N = 3)			16239.06 (1446.63)	18096.45 (2083.61)		57314.29 (7145.93)	61073.73 (7837.33)	
Q4 subgroup of hospitals (N = 3)			18541.10 (2157.90)	20111.56 (3379.42)		56571.17 (6907.53)	71385.67(13184.09)	
Drug costs per case with targeted diseases [RMB], mean (SD)								
Q1 subgroup of hospitals (N = 3)			1897.46 (476.05)	2218.30 (580.55)		11242.36 (4333.24)	11807.70 (5355.19)	
Q2 subgroup of hospitals (N = 3)			2738.45 (807.37)	2415.93 (779.79)		15639.95 (4364.58)	10311.13 (3440.44)	
Q3 subgroup of hospitals (N = 3)			4104.58 (1899.78)	3579.18 (1354.04)		22927.88 (3477.73)	17947.10 (5510.08)	
Q4 subgroup of hospitals (N = 3)			5118.69 (709.41)	3877.32 (1339.61)		23443.73 (6045.40)	22482.90 (5709.41)	
Consumable costs per case with targeted diseases [RMB], mean (SD)								
Q1 subgroup of hospitals (N = 3)			1121.72 (115.35)	2483.72 (940.43)		5.11 (1.61)	4.91 (1.69)	
Q2 subgroup of hospitals (N = 3)			1045.42 (310.01)	2601.85 (1106.94)		7.62 (1.67)	5.19 (1.30)	
Q3 subgroup of hospitals (N = 3)			2409.11 (1101.24)	2594.85 (1138.65)		7.49 (1.32)	6.95 (0.91)	
Q4 subgroup of hospitals (N = 3)			2194.55 (1102.01)	3653.29 (1201.56)		7.75 (1.09)	6.05 (1.22)	
Length of stay per case with targeted diseases [days], mean (SD)								
Q1 subgroup of hospitals (N = 3)			14548.55 (3441.16)	16231.39 (2913.19)		17.41 (2.30)	14.22 (2.32)	
Q2 subgroup of hospitals (N = 3)			15191.99 (1754.77)	18416.43 (3149.26)		18.73 (3.36)	12.89 (2.38)	
Q3 subgroup of hospitals (N = 3)			15537.67 (3611.85)	18811.05 (8735.23)		22.40 (2.43)	19.37 (4.08)	
Q4 subgroup of hospitals (N = 3)			17821.77 (4337.62)	21503.77 (10385.53)		23.76 (1.92)	20.06 (2.67)	

Notes: “Before” represents the overall level from 1st quarter of 2012 to 3rd quarter of 2013, “After” represents the overall level from 4th quarter of 2013 to 3rd quarter of 2020; Quartile 1 (Q1) subgroup represents hospitals with the lowest costs per case at 4th quarter of 2013, Q4 subgroup represents hospitals with the highest costs per case at 4th quarter of 2013

### Statistical analyses

We used quarterly aggregate data to explore the impact of information disclosure on medical costs at hospital level. An interrupted time series with segmented regression analysis was conducted to assess whether there was a difference between cost trends before and after information disclosure concerning diseases. This method is one of the best quasi-experimental study designs available [23], and has frequently been used to evaluate important policy changes even without the availability of a comparison group [24]. The data were divided into two segments: before intervention (disclosing information of diseases) and after intervention. This allowed us to measure changes in medical costs during the study period, including both changes in the levels (immediate change of indicator) and trends (difference between the pre-intervention slope and post-intervention slope) that occurred after information of diseases were disclosed.

The model is as follows:

1 $$a_{b}{{Y}_{t}={\beta }_{0}+{\beta }_{1}{\text{T}}_{\text{t}}+{\beta }_{2}{\text{X}}_{\text{t}}+{\beta }_{3}{\text{X}}_{\text{t}}{\text{T}}_{\text{t}}+{\epsilon}_{t}}$$

where ${Y}_{t}$ represents the outcome variable in each quarter, t was time period (quarter), ${\text{T}}_{\text{t}}$ is a continuous variable modelling each quarter since the first quarter of 2012 (quarter), ${\text{X}}_{\text{t}}$ is a dummy indicator variable (where 0 = before 2013 quarter 4 and 1 = after 2014 quarter 1) and ${\text{X}}_{\text{t}}{\text{T}}_{\text{t}}$ is the interaction term. Hence, ${\beta }_{0}$ is the intercept, ${\beta }_{1}$ is the trend in costs before disclosing information concerning diseases, ${\beta }_{2}$ is the change in costs immediately after information disclosure or the step-change, and ${\beta }_{3}$ is the difference between the pre-disclosure and post-disclosure trends. Accordingly, the post-intervention linear trend is equal to ${\beta }_{1}$+${\beta }_{3}$.

In our interrupted time series model, we did not control for seasonality because seasonality was not observed in the analyzed time series. Sensitivity analyses were performed by adding different combinations of covariates, including hospital type (general or specialist), number of beds, proportion of cases with targeted disease, and fixed effects of seasons to assess the robustness of the model. All analyses were performed using Stata 16.0 with the *itsa* command. Further, 95% CIs were estimated using Newey-West standard errors, which accounted for auto-correlation. We used 5% as the significance level [25].

## Results

### Descriptive statistics

Table 1 summarizes the sample statistics of included hospitals. We identified 14 tertiary public hospitals before and after disease information disclosure. The distribution of hospital type for the two diseases was the same in the pre-and post-disclosure period with eleven (91.67%) general hospitals, and one (8.33%) specialty hospital. In our total sample, the mean beds were 1,630; the mean employees were 3,139; the mean annual discharges per hospital were 88,964; and the mean annual costs per hospital were 3366.73 million RMB. The inpatient cases were 218,928 for the total sample, and 14,246 (106,517) and 13,241(84,924) for the two diseases in the pre-and post-disclosure period, respectively.

### Impact of information disclosure on discharge costs per case

**Fig. 1:**
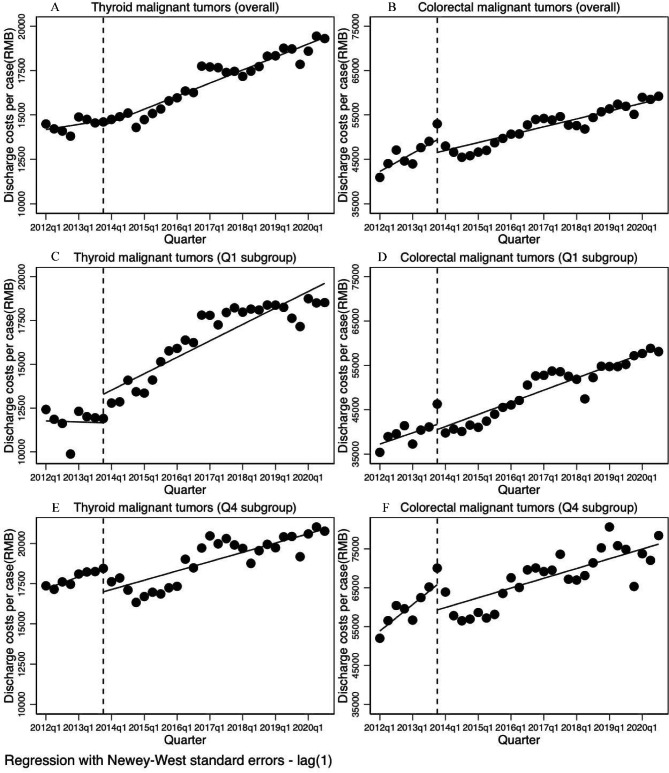
ITS analysis of discharge costs per case

For both thyroid and colorectal malignant tumors, there were decreasing but insignificant step change in discharge costs per case (-284.489 RMB, P = 0.318; -2837.556 RMB, P = 0.059, respectively) when information was disclosed. Ascending change in trend of discharge costs per case after information disclosure were observed in thyroid malignant tumors (111.359 RMB per quarter, P = 0.020). In contrast, discharge costs per case in colorectal malignant tumors presented significant decrease in trend of discharge costs per case (-574.004 RMB per quarter, P = 0.023) after information disclosure (Table 2; Fig. 1A and B).

**Table 2:** Results of interrupted time series showing changes in trend and level change after the information disclosure on diseases

	Constant β0 (SE)	Quarterly trend before information disclosure β1 (SE)	Step change when information disclosed β2 (SE)	Change in trend after information disclosure β3 (SE)
**Thyroid malignant tumors**				
Discharge costs per case [RMB]	14177.888***	72.719	-284.489	111.359*
	(198.326)	(44.240)	(280.417)	(45.479)
Drug costs per case [RMB]	3078.871***	-6.298	-77.198	-21.611*
	(35.062)	(9.509)	(99.450)	(10.451)
Consumable costs per case [RMB]	1466.304***	-11.237	199.174**	89.174***
	(30.700)	(6.710)	(62.717)	(7.508)
Length of stay [days]	7.370***	-0.120***	-0.815***	0.080**
	(0.129)	(0.028)	(0.120)	(0.028)
**Colorectal malignant tumors**				
Discharge costs per case [RMB]	42256.420***	1018.438***	-2837.556	-574.004*
	(1028.788)	(222.443)	(1446.846)	(240.608)
Drug costs per case [RMB]	14288.657***	384.040***	-1732.9479*	-598.438***
	(394.854)	(84.565)	(701.918)	(99.221)
Consumable costs per case [RMB]	14673.734***	5.130	-396.835	222.859***
	(14.654)	(3.705)	(291.280)	(21.144)
Length of stay [days]	19.755***	-0.275***	-1.423***	0.127
	(0.343)	(0.075)	(0.366)	(0.080)

Notes: CI-95% confidence intervals in parentheses; *p < 0.05; **p < 0.01; ***p < 0.001; SE: Standard Errors

As shown in Table 3, discharge costs per case varied significantly across quartiles. For thyroid malignant tumors, Q1 hospitals with the lowest baseline costs demonstrated significant increase in both step change (1629.251 RMB, P = 0.019) and change in trend (250.365 RMB per quarter, P = 0.018) of costs per case, whereas Q4 hospitals with the highest baseline costs experienced significant decrease in step change (-1504.189 RMB, P = 0.003). For colorectal malignant tumors, there were neither significant step change nor change in trend of costs per case in Q1 hospitals, while significant decrease in both step change (-6511.650 RMB, P = 0.024) and change in trend (-1076.060 RMB per quarter, P = 0.006) were found in Q4 hospitals with the highest baseline costs (Fig. 1C-F).

**Table 3:** Results of interrupted time series showing changes in trend and level change according to quartiles of hospital discharge costs per case after the information disclosure

	Thyroid malignant tumors				Colorectal malignant tumors			
	Q1	Q2	Q3	Q4	Q1	Q2	Q3	Q4
Constant β0 (SE)	11764.385***	13880.309***	16176.986***	17172.461***	37242.227***	39809.620***	55186.052***	53864.407***
	(450.637)	(135.857)	(378.180)	(88.921)	(1005.794)	(1756.612)	(1033.777)	(1342.396)
Quarterly trend before information disclosure β1 (SE)	-15.508	117.078**	-1.335	189.307***	635.738**	1198.890**	883.358***	1704.025***
	(97.772)	(36.776)	(76.513)	(22.839)	(219.964)	(365.307)	(237.346)	(312.207)
Step change when information disclosed β2 (SE)	1629.251*	-735.532*	124.565	-1504.189**	-1173.341	-4037.882*	-1056.318	-6511.650*
	(656.360)	(283.823)	(518.349)	(463.237)	(1424.749)	(1826.916)	(1363.632)	(2748.788)
Change in trend after information disclosure β3 (SE)	250.365*	56.176	151.906	-44.700	48.350	-807.3128*	-892.494**	-1076.060**
	(100.377)	(37.522)	(79.571)	(30.908)	(228.392)	(382.820)	(254.425)	(361.946)

Notes: CI-95% confidence intervals in parentheses; *p < 0.05; **p < 0.01; ***p < 0.001; SE: Standard Errors;Quartile 1 (Q1) represents the lowest discharge costs per case and Q4 represents the highest discharge costs per case

### Impact of information disclosure on drug costs per case

Immediate decrease in drug costs per case after information disclosure were observed but only significant in colorectal malignant tumors (-1732.948 RMB, P = 0.019). However, both thyroid (-21.611 RMB per quarter, P = 0.047) and colorectal malignant tumors (-598.438 RMB per quarter, P < 0.001) presented significant descending changes in trend of drug costs per case (Table 2).

There was significant immediate increase in drug costs per case for thyroid malignant tumors among Q1 hospitals (175.500 RMB, P = 0.010), as well as significant decrease in both step change (-849.364 RMB, P < 0.001) and change in trend (-119.684 RMB per quarter, P < 0.001) among Q4 hospitals. For colorectal malignant tumors, all 4 subgroups experienced significant descending change in trend of drug costs per case, among which Q4 hospitals demonstrated the largest decrease (-1101.486 RMB per quarter, P < 0.001) (supplementary Table 2).

### Impact of information disclosure on consumable costs per case

As shown in Table 2, consumable costs per case for thyroid malignant tumors experienced significant increase in both step change (199.174 RMB, P = 0.003) and change in trend (89.174 RMB per quarter, P < 0.001) after information disclosure. For colorectal malignant tumors, consumable costs per case demonstrated significant increasing change in trend (222.859 RMB per quarter, P < 0.001) after information disclosure.

In terms of step change in consumable costs per case, Q1 hospitals underwent immediate increase for thyroid malignant tumors (420.973 RMB, P = 0.002), Q4 hospitals presented immediate decrease for colorectal malignant tumors (-3893.801 RMB, P < 0.001), while Q2 and Q3 hospitals experienced insignificant step change. Ascending change in trend of consumable costs per case were observed in both thyroid and colorectal malignant tumors across the four subgroups (supplementary Table 3).

### Impact of information disclosure on length of stay

Immediately after the performance information was disclosed, significant decrease in length of stay were found in both thyroid (-0.815, P < 0.001) and colorectal malignant tumors (-1.423, P < 0.001). Increase in trend of length of stay were observed in both diseases but only significant in thyroid malignant tumors (0.080, P = 0.008) (Table 2).

For most of the subgroups, neither step change nor change in trend were found in both thyroid and colorectal malignant tumors. Notably, Q4 hospitals experienced immediate decrease in length of stay (-1.656, P = 0.028) among cases with colorectal malignant tumors (supplementary Table 4).

**Table 4:** Results of sensitivity analysis showing changes in trend and level change of discharge costs per case after the information disclosure

	Model1	Model2	Model3	Model4	Model5
**Thyroid malignant tumors**					
Constant β0 (SE)	14190.437***	1788.894	1070.168	1725.670	3293.394
	(190.756)	(8994.661)	(9658.048)	(8853.679)	(9731.808)
Quarterly trend before information disclosure β1 (SE)	81.019	63.961	67.612	68.327	60.547
	(41.936)	(41.590)	(45.064)	(41.395)	(45.874)
Step change when information disclosed β2 (SE)	-299.431	-26664.581*	-25615.156*	-27630.185*	-29002.501*
	(255.339)	(10283.471)	(10368.810)	(12019.475)	(12749.264)
Change in trend after information disclosure β3 (SE)	102.750*	123.984*	119.237*	129.068*	135.720*
	(43.270)	(48.531)	(48.990)	(56.223)	(59.694)
Season (SE)					
Quarter 2	60.146	-	54.892	-	141.186
	(163.675)	-	(87.740)	-	(116.260)
Quarter 3	-116.192	-	-51.382	-	109.029
	(185.103)	-	(115.090)	-	(163.882)
Quarter 4	-150.040	-	-232.028	-	-38.931
	(254.194)	-	(178.239)	-	(202.170)
Specialist hospital (SE)	-	-1461.337*	-1466.718*	7895.359	7896.487
	-	(551.176)	(551.149)	(4761.454)	(4807.687)
Number of beds (SE)	-	-0.013	-0.027	0.284	0.289
	-	(0.671)	(0.683)	(0.622)	(0.637)
Proportion of cases with targeted diseases (SE)	-	-	-	-179.659	-179.637
	-	-	-	(96.934)	(97.733)
**Colorectal malignant tumors**					
Constant β0 (SE)	42121.516***	-183075.660***	-188307.540***	-151438.030***	-148564.940***
	(1024.411)	(36358.173)	(34428.331)	(32439.770)	(29917.177)
Quarterly trend before information disclosure β1 (SE)	972.0792***	1155.438***	1183.589***	1036.589***	1022.861***
	(229.902)	(167.464)	(166.819)	(151.778)	(142.337)
Step change when information disclosed β2 (SE)	-2681.814	138755.910**	144957.190**	108078.170*	106229.740*
	(1534.924)	(43086.709)	(37490.546)	(41044.968)	(37222.896)
Change in trend after information disclosure β3 (SE)	-528.164*	-653.446**	-682.473**	-508.969*	-499.510*
	(239.472)	(206.374)	(179.317)	(195.009)	(176.373)
Season (SE)					
Quarter 2	26.152	-	-1222.456	-	276.935
	(654.642)	-	(871.206)	-	(772.597)
Quarter 3	649.018	-	-388.033	-	190.861
	(844.118)	-	(800.596)	-	(640.992)
Quarter 4	567.514	-	-920.765	-	-477.754
	(1019.975)	-	(729.973)	-	(758.235)
Specialist hospital (SE)	-	-12038.855	-12073.224**	14513.611*	14552.010*
	-	(3580.016)	(3611.290)	(5433.259)	(5475.528)
Number of beds (SE)	-	-6.757	-6.834	-3.614	-3.628
	-	(5.103)	(5.180)	(2.806)	(2.866)
Proportion of cases with targeted diseases (SE)	-	-	-	-1358.079***	1360.466***
	-	-	-	(289.302)	(289.652)

Notes: CI-95% confidence intervals in parentheses; *p < 0.05; **p < 0.01; ***p < 0.001; SE: Standard Errors; Model 1 adjusted season fixed effects; Model 2 adjusted hospital type and number of beds per quarter; Model 3 adjusted hospital type, number of beds per quarter, and season fixed effects; Model 4 adjusted hospital type, number of beds per quarter, and proportion of cases with targeted disease of total cases per hospital; Model 5 adjusted hospital type, number of beds per quarter, proportion of cases with targeted disease of total cases per hospital and season fixed effect

### Sensitivity analysis

The sensitivity analysis in which we adjusted season fixed effects, hospital type, number of beds and proportion of cases with targeted disease gave similar but more precise results compared with the primary analysis. The findings produced smaller step changes, and the directions of pre-disclosure and post-disclosure trends were consistent with those observed in the main analysis. However, more statistical significance in changes was found compared with in the initial analysis (Table 4 and supplementary Tables 5, supplementary Tables 6, supplementary Tables 7, and supplementary Fig. 2).

## Discussion

In this study, disclosure of information among public hospitals in Shanghai had effect on costs per case in each hospital. For malignant thyroid and colorectal tumors, the discharge costs per case presented an insignificant downward trend immediately upon information disclosure, but a significant different trend change subsequently. Meanwhile, the results showed that information disclosure resulted in a significant increase in the quarterly trend of discharge costs per case among low-cost hospitals, whereas a slight decrease in the discharge cost trend was observed among high-cost hospitals.

It is important to note that significant declines in both drug expenditures and length of stay were detected. In this study, the reduction of discharge costs per case may be related to reduction in unnecessary drug usage or excessive hospitalization. Moreover, the reduction in the consumable costs per case is not yet demonstrated. In addition, in the fourth quarter of 2013, the main policy that affected the health cost was the disclosure of costs on diseases, and there was no other policy that directly affected the costs. Therefore, the time displacement of the introduction of different reform measures, to some extent, eliminates the interference of other policy factors on information disclosure of this study.

Our results provide evidence that information disclosure did indeed generate effects partially consistent with the intended purposes. The findings suggest that the cost change was primarily driven by disease information, which was presented in the form of a series of cost and efficiency indicators. For public reporting what matters most is that hospital managers are shown to meet acceptable performance standards [26]. For example, an indicator that shows a hospital is a high-cost outlier in medical service provides a clear indication that this dimension requires attention. It could be said that the very availability of this homogenous information made various hospitals managers to compare their costs with those of their counterparts, and in so doing, maintain professional status. As opposed to the studies performed on patients, little information is available from the literature regarding the effectiveness of information disclosure on healthcare provider. Larger, adequately powered studies that are designed to assess the impact of information disclosure on medical costs are required to confirm our positive finding.

In addition, similar information reported high- and low-cost hospitals by rank-ordering them based on costs. This can have a powerful effect on reputation, as high-cost hospitals can easily imitate the disclosure behavior of low-cost hospitals, as we observed in this study. The findings indicated that making interhospital information public stimulated medical cost reduction in low-cost hospitals. Concern for public image appeared to be a key motivator for hospitals’ medical cost change efforts [20, 27]. In general, hospitals performing well after information disclosure will continue to maintain professional or institutional leading advantages. However, hospitals performing really poorly will experience an existential crisis, which in turn will force them to engage in a wide range of increased improvement efforts immediately and invest in building a reputation after information disclosure. Thus, the effect of disclosing interhospital performance information on hospital behavior is important to understand the spillover effects of information disclosure and thus merits our research. We believe that the reason for this spillover effect is the improvement of cognition and identity. The implication is that information disclosure, if implemented in a form that is reasonably designed and is relatively scientific, can have a strong positive impact on low-cost hospitals. As such, whether this cost change behavior of hospitals with different ratings is competition driven, or somehow driven by hospital management decisions remains to be determined in future research.

It is also worth noting that the findings in this study were entirely driven by supply-side responses to performance information. Performance information generally has decision value, and as stated by Narayanan and Davila [28]: “Most firms collect a plethora of information for belief revision, even though only a few signals are directly linked to incentives.” Most proponents of the disclosure of healthcare performance information believe that making this information public will enhance the decision-making of providers and encourage market-based discipline and reform [29, 30]. Our results also support the notion that making performance information public between hospitals can have certain decision-making value for hospitals. It is further possible that the positioning of the hospital in the medical care field based on the performance report affects its status, respect, etc., as the face value “assumes a particular importance” in East Asia. Face plays the role of a “social interactional identity,” directing a hospital’s verbal and non-verbal behaviors that “protect/save self-face” [31]. Organizations desire recognition as a motivator of consequent feedback-seeking activities. The study by Hwang et al. arrived at similar conclusions and has suggested that in collectivist culture, face awareness is more obvious, and brand-name products can improve the organization’s social self and peer recognition [32]. Therefore, executive leaders have an inherent motivation to improve and consider them and the measures that underlie them while making medical decisions. At the same time, Hibbard et al. also noted that hospitals with public reporting programs engaged in more quality improvement activities and were more likely to have improved outcomes than their counterparts [13]. However, a greater number of high-quality studies are required to verify the effects of information disclosure.

To the best of our knowledge, this is the first study to evaluate the effect of information disclosure on medical costs among medical organizations. We believe that our findings will not only have a strong positive impact on medical cost change efforts but it will also enable the formation of an effective information disclosure form, which in turn could help improve and enhance the supervision and governance among medical institutions in Shanghai. Moreover, this form of information disclosure, once fully established, can give provider-specific performance information within the industry. Further, the decision making by hospital can be better informed. Finally, our study can serve as useful background research for further large-scale, multicenter studies and for research after comprehensive medical information disclosure.

Our study does have limitations that need to be acknowledged. First, in our analysis, we examined changes in costs per case over approximately 27 quarters after the disclosure of disease information. Further high-quality studies should focus on evaluating changes in efficiency and quality of services provided that we were unable to observe with the current data. Further, more importantly, the degree to which the observed increased quality and efficiency improvement efforts yield actual improvements in outcomes should be observed. Second, we could not examine other effects of hospital-level changes that may be important, such as, different personal preferences of executives at the hospital level, different cultural backgrounds of hospitals, and different strategies used to confirm the development goals. Examination of these issues was beyond the scope of the current study. These will be critical topics for future research. Third, although this research cannot draw a firm causal link between information disclosure and outcomes in light of a non-experimental design, an interrupted time series design was used to study time trends in an adjusted analysis for important prognostic factors and for exploring the impact of information disclosure on outcomes. We did not identify significant changes in healthcare-related policies in fourth quarter of 2013, which reduces the concern that we may be misattributing changes in outcomes to factors other than the intervention in question. Finally, because of the limitation of sample selection, the results of our study can only represent the cities with comparable sample GDP levels. Future studies are needed to disentangle the impact of these policies.

## Conclusion

In conclusion, information disclosure resulted in a significant increase in the quarterly trend in discharge costs per case among low-cost hospitals, whereas a slight reduction in the costs among high-cost hospitals was seen. Our findings support the continued disclosure of medical information, improvements in the disclosure form, and enlargement of the public reporting systems in China, given its potential for triggering changes in healthcare costs. In future studies, evaluating the types of information or presentation methods that are effective in making medical decisions and ultimately providing care will help verify our findings.

## Electronic supplementary material

Below is the link to the electronic supplementary material.

Supplementary Material 1

## Data Availability

The datasets generated or analyzed during the current study are not publicly available due confidentiality policies but are available from the corresponding author on reasonable request.
