# Supplementary material for: Does information disclosure among public hospitals stimulate medical cost change efforts? A pilot study in Shanghai
Source: BMC Health Serv Res. 2023 May 24;23:531. doi: 10.1186/s12913-023-09510-8 (PMC10210391; doi:10.1186/s12913-023-09510-8)
Supplement: Supplementary file 1 — Supplementary Material 1 [file 12913_2023_9510_MOESM1_ESM.docx]

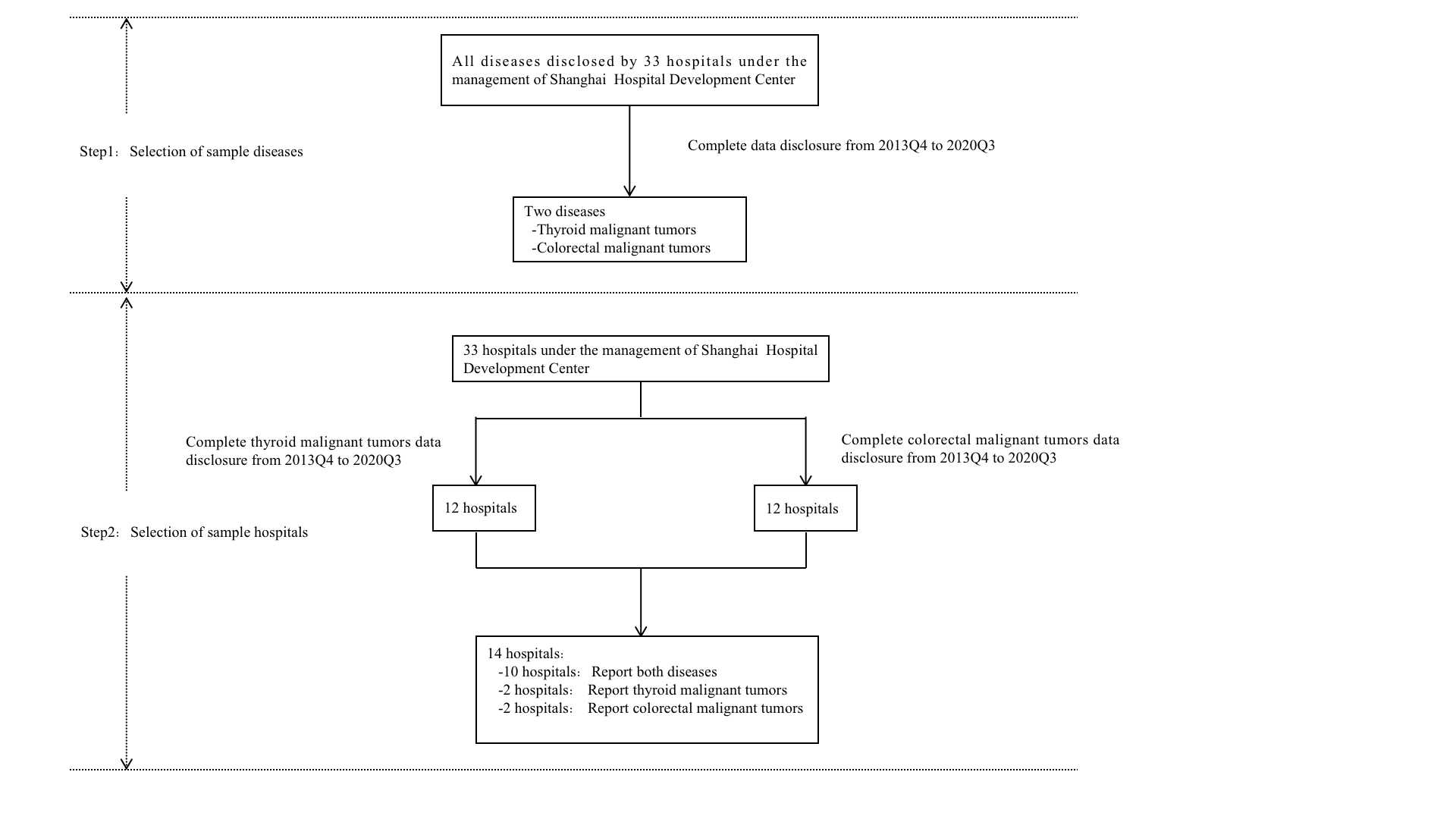


Supplementary figure 1 Flow chart of sample disease and hospital selection

Supplementary table 1 The available variables in the report cards

| Variable | What cost measure captures | Whether included in characteristic description | Whether included in multivariate regression |
| --- | --- | --- | --- |
| **Hospital characteristics** | | | |
| Hospital type | General hospital or specialized hospital | Yes | Yes, as covariate |
| Number of beds | Fixed actual beds at the end of each quarter | Yes | Yes, as covariate |
| Number of employees | Total number of employees at the end of each quarter | Yes | Yes, as covariate |
| Annual discharges per hospital | Number of hospital discharges per hospital per year | Yes | No |
| Annual discharge costs per hospital | Hospital charges of all inpatients in the unit of hospitalization times | Yes | No |
| Length of stay | Average length of hospital stay for each discharged person | Yes | No |
| **Inpatient characteristics** | | | |
| Cases with targeted diseases | Number of hospital discharges with targeted diseases | Yes | No |
| Proportion of cases with targeted diseases | Proportion of the number of inpatients in all hospitals publishing the targeted disease | Yes | Yes, as covariate |
| Discharge costs per case with targeted diseases | Average value of hospital charges of all inpatients in the unit of hospitalization times | Yes | Yes, as outcome |
| Proportion of discharge costs on targeted diseases | Proportion of hospital charges for the disease in all hospitals publishing the targeted disease | Yes | Yes, as covariate |
| Drug costs per case with targeted diseases | Average cost of medicines per inpatient hospital stay | Yes | Yes, as outcome |
| Consumable costs per case with targeted diseases | Average cost of consumables per inpatient hospital stay | Yes | Yes, as outcome |
| Length of stay per case with targeted diseases | Average length of stay per inpatient hospital stay | Yes | Yes, as outcome |

Data resource：Hospital-level performance report issued by Shanghai Hospital Development Center.

Supplementary table 2 Results of interrupted time series showing changes in trend and level change according to quartiles of hospital drug costs per case after the information disclosure

|  | Thyroid malignant tumors | | | | Colorectal malignant tumors | | | |
| --- | --- | --- | --- | --- | --- | --- | --- | --- |
|  | Q1 | Q2 | Q3 | Q4 | Q1 | Q2 | Q3 | Q4 |
| Constant  β0 (SE) | 1929.608*** | 2797.196*** | 4128.006*** | 5082.066*** | 9267.867*** | 14087.864*** | 21900.320*** | 23416.503*** |
|  | 36.919 | 47.885 | 48.892 | 103.436 | 232.612 | 783.280 | 445.138 | 642.724 |
| Quarterly trend before  information disclosure  β1 (SE) | -6.835 | 8.497 | -65.167*** | 41.746 | 173.109** | 438.611** | 372.163*** | 780.185*** |
|  | （8.466） | （13.059） | （10.816） | （23.711） | （51.650） | （159.708） | （99.297） | （144.205） |
| Step change when information disclosed  β2 (SE) | 175.500** | -87.644 | 366.401 | -849.364*** | 5.593 | -3171.335** | -1417.532 | -2603.277 |
|  | （63.569） | （98.483） | （377.445） | （219.453） | （401.449） | （1145.448） | （817.100） | （1442.207） |
| Change in trend after  information disclosure  β3 (SE) | 13.766 | -41.104** | 31.895 | -119.684*** | -164.281** | -710.841*** | -795.208*** | -1101.486*** |
|  | （9.489） | （13.716） | （20.765） | （27.471） | （59.351） | （177.114） | （113.929） | （167.983） |

Notes: CI-95% confidence intervals in parentheses; *p < 0.05; **p < 0.01; ***p < 0.001; SE: Standard Errors；Quartile 1 (Q1) represents the lowest discharge costs per case and Q4 represents the highest discharge costs per case.

Supplementary table 3 Results of interrupted time series showing changes in trend and level change according to quartiles of hospital consumable costs per case after the information disclosure

|  | Thyroid malignant tumors | | | |  | Colorectal malignant tumors | | | |
| --- | --- | --- | --- | --- | --- | --- | --- | --- | --- |
|  | Q1 | Q2 | Q3 | Q4 |  | Q1 | Q2 | Q3 | Q4 |
| Constant  β0 (SE) | 1207.400*** | 1260.876*** | 2319.757*** | 1736.393*** |  | 13338.079*** | 14808.003*** | 15593.840*** | 17871.693*** |
|  | (22.656) | (53.361) | (56.069) | (32.989) |  | (32.172) | (44.421) | (101.472) | (34.373) |
| Quarterly trend before information disclosure β1 (SE) | -18.376** | -11.089 | 12.629 | 1.805 |  | 9.042 | -6.927 | 9.817 | -11.392 |
|  | (5.161) | (10.249) | (12.865) | (7.832) |  | (7.176) | (8.784) | (22.637) | (7.756) |
| Step change when information disclosed  β2 (SE) | 420.973** | 178.500 | 132.141 | 94.608 |  | -294.411 | -104.169 | 1193.224 | -3893.801*** |
|  | (126.329) | (96.023) | (101.069) | (136.421) |  | (175.994) | (375.242) | (1497.078) | (859.534) |
| Change in trend after information disclosure β3 (SE) | 86.807*** | 101.829*** | -12.515 | 113.329*** |  | 204.898*** | 261.678*** | 107.808 | 283.995** |
|  | (11.700) | (11.493) | (14.216) | (13.168) |  | (16.676) | (29.387) | (70.247) | (79.310) |
| Notes: CI-95% confidence intervals in parentheses; *p < 0.05; **p < 0.01; ***p < 0.001; SE: Standard Errors；Quartile 1 (Q1) represents the lowest discharge costs per case and Q4 represents the highest discharge costs per case. | | | | | | | | | |

Supplementary table 4 Results of interrupted time series showing changes in trend and level change according to quartiles of hospital length of stay after the information disclosure

|  | Thyroid malignant tumors | | | |  | Colorectal malignant tumors | | | |
| --- | --- | --- | --- | --- | --- | --- | --- | --- | --- |
|  | Q1 | Q2 | Q3 | Q4 |  | Q1 | Q2 | Q3 | Q4 |
| Constant  β0 (SE) | 4.493*** | 8.190*** | 7.877*** | 7.631*** |  | 17.642*** | 19.471*** | 22.534*** | 25.086*** |
|  | (0.127) | (0.109) | (0.208) | (0.134) |  | (0.426) | (0.140) | (0.629) | (0.514) |
| Quarterly trend before information disclosure β1 (SE) | -0.053 | -0.149*** | -0.098* | -0.104** |  | -0.274** | -0.334*** | -0.093 | -0.278* |
|  | (0.027) | (0.027) | (0.047) | (0.029) |  | (0.095) | (0.039) | (0.167) | (0.109) |
| Step change when information disclosed  β2 (SE) | -0.053 | -1.561*** | 0.652* | 0.239 |  | 0.133 | -2.959*** | -0.392 | -1.656* |
|  | (0.113) | (0.204) | (0.250) | (0.136) |  | (0.339) | (0.461) | (0.695) | (0.716) |
| Change in trend after information disclosure β3 (SE) | 0.057 | 0.104** | 0.037 | 0.014 |  | 0.128 | 0.216*** | -0.095 | 0.121 |
|  | (0.028) | (0.029) | (0.047) | (0.030) |  | (0.096) | (0.049) | (0.178) | (0.115) |
| Notes: CI-95% confidence intervals in parentheses; *p < 0.05; **p < 0.01; ***p < 0.001; SE: Standard Errors；Quartile 1 (Q1) represents the lowest discharge costs per case and Q4 represents the highest discharge costs per case. | | | | | | | | | |

Supplementary table 5 Results of sensitivity analysis showing changes in trend and level change of drug costs per case after the information disclosure

|  | Model1 | Model2 | Model3 | Model4 | Model5 |
| --- | --- | --- | --- | --- | --- |
| **Thyroid malignant tumors** | | | | | |
| constant  β0 (SE) | 3058.164*** | 7856.355 | 8784.549 | 7825.643 | 9871.593 |
|  | (51.475) | (5409.176) | (5922.232) | (5352.70) | (5970.120) |
| Quarterly trend before  information disclosure  β1 (SE) | -10.836 | -19.024 | -23.570 | -16.903 | -27.024 |
|  | (14.430) | (22.797) | (25.539) | (22.378) | (25.734) |
| Step change when information disclosed  β2 (SE) | -48.328 | 2362.770 | 1482.735 | 1893.718 | -173.504 |
|  | (111.791) | (6146.219) | (6363.935) | (6335.299) | (6736.210) |
| Change in trend after  information disclosure  β3 (SE) | -17.547 | -10.572 | -6.370 | -8.102 | 1.690 |
|  | (14.749) | (29.260) | (30.308) | (30.021) | (31.938) |
| Season (SE) | | | | | |
| Quarter 2 | 56.671 | - | 19.695 | - | 61.888 |
|  | (70.993) | - | (66.049) | - | (73.183) |
| Quarter 3 | 63.533 | - | 63.717 | - | 142.149 |
|  | (95.831) | - | (75.667) | - | (86.793) |
| Quarter 4 | -0.160 | - | 12.402 | - | 106.817 |
|  | (90.457) | - | (100.589) | - | (101.224) |
| Specialist hospital (SE) | - | -445.394 | -443.893 | 4099.723* | 4134.236* |
|  | - | (377.680) | (379.422) | (1711.516) | (1719.618) |
| Number of beds (SE) | - | -0.239 | -0.235 | -0.094 | -0.081 |
|  | - | (0.526) | (0.534) | (0.513) | (0.520) |
| Proportion of cases with targeted diseases (SE) | - | - | - | -87.271* | ‘-87.833* |
|  | - | - | - | (33.148) | (33.237) |
| **Colorectal malignant tumors** | | | | | |
| Constant  β0 (SE) | 14242.887*** | -71115.779** | -69006.641** | -52275.294** | -45349.985** |
|  | (391.752) | (18021.324) | (15708.134) | (14677.353) | (12170.837) |
| Quarterly trend before  information disclosure  β1 (SE) | 347.382*** | 446.231*** | 437.341*** | 375.456*** | 341.668*** |
|  | (88.428) | (82.448) | (75.646) | (70.836) | (60.792) |
| Step change when information disclosed  β2 (SE) | -1549.142 | 137570.300*** | 135983.430*** | 119301.430*** | 112931.050*** |
|  | (775.804) | (29103.223) | (26931.384) | (24863.898) | (23689.347) |
| Change in trend after  information disclosure  β3 (SE) | -564.076*** | -645.087*** | -637.390*** | -559.050*** | -528.481*** |
|  | (97.730) | (137.447) | (127.113) | (116.475) | (110.845) |
| Season (SE) | | | | | |
| Quarter 2 | -21.327 | - | -699.886 | - | 192.621 |
|  | (438.099) | - | (411.748) | - | (462.474) |
| Quarter 3 | 513.217 | - | 125.861 | - | 470.446 |
|  | (633.815) | - | (473.373) | - | (401.819) |
| Quarter 4 | 106.438 | - | -297.686 | - | -33.985 |
|  | (531.702) | - | (403.519) | - | (411.204) |
| Specialist hospital (SE) | - | -4760.368 | -4768.689** | 11051.858* | 11079.893* |
|  | - | (1442.349) | (1444.887) | (4091.755) | (4133.487) |
| Number of beds (SE) | - | -2.938 | -2.956 | -1.066 | -1.047 |
|  | - | (2.961) | (3.004) | (2.210) | (2.249) |
| Proportion of cases with targeted diseases (SE) | - | - | - | -808.748** | -809.813** |
|  | - | - | - | (192.252) | (193.599) |

Notes: CI-95% confidence intervals in parentheses; *p < 0.05; **p < 0.01; ***p < 0.001; SE: Standard Errors；Model 1 adjusted season fixed effects. Model 2 adjusted hospital type and number of beds per quarter. Model 3 adjusted hospital type, number of beds per quarter, and season fixed effects. Model 4 adjusted hospital type, number of beds per quarter, and proportion of cases with targeted disease of total cases per hospital. Model 5 adjusted hospital type, number of beds per quarter, proportion of cases with targeted disease of total cases per hospital and season fixed effect.

Supplementary table 6 Results of sensitivity analysis showing changes in trend and level change of consumable costs per case after the information disclosure

|  | Model1 | Model2 | Model3 | Model4 | Model5 |
| --- | --- | --- | --- | --- | --- |
| **Thyroid malignant tumors** | | | | | |
| Constant  β0 (SE) | 1670.646*** | 2219.999*** | 2209.116*** | 2254.215*** | 2234.226*** |
|  | (290.648) | (389.596) | (389.320) | (416.480) | (415.194) |
| Quarterly trend before information disclosure β1 (SE) | -6.142 | -2.575 | -4.040 | -2.495 | -4.118 |
|  | (6.559) | (6.181) | (7.716) | (6.045) | (7.886) |
| Step change when information disclosed  β2 (SE) | -366.584* | -354.904* | -328.925* | -352.380* | -327.804 |
|  | (151.912) | (140.351) | (145.179) | (138.579) | (143.729) |
| Change in trend after information disclosure β3 (SE) | 75.664*** | 75.589*** | 76.255*** | 75.802*** | 76.557*** |
|  | (19.025) | (16.126) | (17.886) | (16.000) | (17.838) |
| Season (SE) |  |  |  |  |  |
| Quarter 2 | 130.794 |  | 99.049 |  | 99.870 |
|  | (67.146) |  | (60.273) |  | (59.877) |
| Quarter 3 | 57.036 |  | 20.453 |  | 22.403 |
|  | (51.907) |  | (47.154) |  | (47.192) |
| Quarter 4 | -49.306 |  | -89.844* |  | -87.423* |
|  | (45.173) |  | (40.849) |  | (42.973) |
| Specialist hospital (SE) |  | -155.361 | -156.539 | 44.195 | -2.527 |
|  |  | (293.312) | (293.228) | (858.291) | (876.842) |
| Number of beds (SE) |  | -0.353** | -0.357** | -0.358** | -0.360** |
|  |  | (0.119) | (0.116) | (0.113) | (0.111) |
| Proportion of cases with targeted diseases (SE) |  |  |  | -3.910 | -3.014 |
|  |  |  |  | (15.615) | (15.939) |
| **Colorectal malignant tumors** | | | | | |
| Constant  β0 (SE) | 15721.838*** | 17713.087*** | 17769.807*** | 21344.909*** | 21405.053*** |
|  | (1131.124) | (1573.771) | (1708.417) | (2773.012) | (2645.991) |
| Quarterly trend before information disclosure β1 (SE) | 45.748 | 8.240 | 61.431 | -26.292 | 21.298 |
|  | (36.340) | (12.393) | (39.318) | (37.356) | (23.441) |
| Step change when information disclosed  β2 (SE) | -2496.521** | -2350.995** | -2290.221** | -2431.966** | -2352.384** |
|  | (827.907) | (775.014) | (697.231) | (805.493) | (737.040) |
| Change in trend after information disclosure β3 (SE) | 214.330** | 252.500*** | 204.676** | 297.495*** | 254.516*** |
|  | (68.929) | (64.467) | (66.155) | (80.335) | (71.679) |
| Season (SE) |  |  |  |  |  |
| Quarter 2 | 19.449 |  | -70.915 |  | 324.880* |
|  | (200.920) |  | (241.858) |  | (131.960) |
| Quarter 3 | -623.014 |  | -741.561 |  | -680.774 |
|  | (492.000) |  | (535.319) |  | (423.495) |
| Quarter 4 | 298.257 |  | 154.559 |  | 144.103 |
|  | (756.132) |  | (739.459) |  | (737.285) |
| Specialist hospital (SE) |  | -1739.731 | -1756.228 | 6710.806 | 6994.854 |
|  |  | (1431.888) | (1445.487) | (4723.973) | (4632.610) |
| Number of beds (SE) |  | -1.239 | -1.279 | -1.068 | -1.093 |
|  |  | (0.670) | (0.686) | (0.630) | (0.653) |
| Proportion of cases with targeted diseases (SE) |  |  |  | -450.846 | -466.671 |
|  |  |  |  | (261.815) | (255.580) |
| Notes: CI-95% confidence intervals in parentheses; *p < 0.05; **p < 0.01; ***p < 0.001; SE: Standard Errors; Model 1 adjusted season fixed effects; Model 2 adjusted hospital type and number of beds per quarter; Model 3 adjusted hospital type, number of beds per quarter, and season fixed effects; Model 4 adjusted hospital type, number of beds per quarter, and proportion of cases with targeted disease of total cases per hospital; Model 5 adjusted hospital type, number of beds per quarter, proportion of cases with targeted disease of total cases per hospital and season fixed effect. | | | | | |

Supplementary table 7 Results of sensitivity analysis showing changes in trend and level change of length of stay after the information disclosure

|  | Model1 | Model2 | Model3 | Model4 | Model5 |
| --- | --- | --- | --- | --- | --- |
| **Thyroid malignant tumors** | | | | | |
| Constant  β0 (SE) | 7.335*** | 7.543*** | 7.583*** | 7.918*** | 7.934*** |
|  | (0.562) | (0.847) | (0.853) | (0.889) | (0.896) |
| Quarterly trend before information disclosure β1 (SE) | -0.079** | -0.084** | -0.077** | -0.083** | -0.078** |
|  | (0.027) | (0.029) | (0.028) | (0.030) | (0.027) |
| Step change when information disclosed  β2 (SE) | -0.152 | -0.175 | -0.130 | -0.147 | -0.113 |
|  | (0.434) | (0.450) | (0.451) | (0.427) | (0.421) |
| Change in trend after information disclosure β3 (SE) | 0.015 | 0.022 | 0.015 | 0.024 | 0.019 |
|  | (0.036) | (0.038) | (0.037) | (0.034) | (0.032) |
| Season (SE) |  |  |  |  |  |
| Quarter 2 | 0.033 |  | 0.015 |  | 0.025 |
|  | (0.050) |  | (0.050) |  | (0.048) |
| Quarter 3 | -0.073 |  | -0.092 |  | -0.068 |
|  | (0.044) |  | (0.051) |  | (0.062) |
| Quarter 4 | -0.108 |  | -0.130 |  | -0.099 |
|  | (0.063) |  | (0.073) |  | (0.069) |
| Specialist hospital (SE) |  | -0.080 | -0.087 | 2.038 | 1.974 |
|  |  | (0.470) | (0.470) | (2.082) | (2.087) |
| Number of beds [thousand] (SE) |  | -0.145 | -0.163 | -0.199 | -0.211 |
|  |  | (0.261) | (0.264) | (0.271) | (0.274) |
| Proportion of cases with targeted diseases (SE) |  |  |  | -0.042 | -0.040 |
|  |  |  |  | (0.041) | (0.041) |
| **Colorectal malignant tumors** | | | | | |
| Constant  β0 (SE) | 21.792*** | 22.039*** | 22.453*** | 22.733*** | 22.923*** |
|  | (1.104) | (1.611) | (1.692) | (2.010) | (2.060) |
| Quarterly trend before information disclosure β1 (SE) | -0.202** | -0.237*** | -0.198** | -0.243*** | -0.202*** |
|  | (0.066) | (0.069) | (0.071) | (0.061) | (0.062) |
| Step change when information disclosed  β2 (SE) | -1.675* | -1.713 | -1.623 | -1.722 | -1.624 |
|  | (0.830) | (0.879) | (0.874) | (0.893) | (0.883) |
| Change in trend after information disclosure β3 (SE) | 0.058 | 0.091 | 0.055 | 0.099* | 0.061 |
|  | (0.057) | (0.059) | (0.061) | (0.049) | (0.049) |
| Season (SE) |  |  |  |  |  |
| Quarter 2 | -0.808*** |  | -0.834*** |  | -0.791*** |
|  | (0.200) |  | (0.224) |  | (0.190) |
| Quarter 3 | -0.503** |  | -0.534** |  | -0.531** |
|  | (0.176) |  | (0.203) |  | (0.198) |
| Quarter 4 | -0.247 |  | -0.283 |  | -0.289 |
|  | (0.145) |  | (0.162) |  | (0.160) |
| Specialist hospital (SE) |  | -3.606** | -3.645** | -2.134 | -2.650 |
|  |  | (1.264) | (1.272) | (2.266) | (2.233) |
| Number of beds [thousand] (SE) |  | -0.146 | -0.238 | -0.153 | -0.249 |
|  |  | (0.527) | (0.557) | (0.513) | (0.541) |
| Proportion of cases with targeted diseases (SE) |  |  |  | -0.079 | -0.054 |
|  |  |  |  | (0.136) | (0.132) |
| Notes: CI-95% confidence intervals in parentheses; *p < 0.05; **p < 0.01; ***p < 0.001; SE: Standard Errors; Model 1 adjusted season fixed effects; Model 2 adjusted hospital type and number of beds per quarter; Model 3 adjusted hospital type, number of beds per quarter, and season fixed effects; Model 4 adjusted hospital type, number of beds per quarter, and proportion of cases with targeted disease of total cases per hospital; Model 5 adjusted hospital type, number of beds per quarter, proportion of cases with targeted disease of total cases per hospital and season fixed effect. | | | | | |


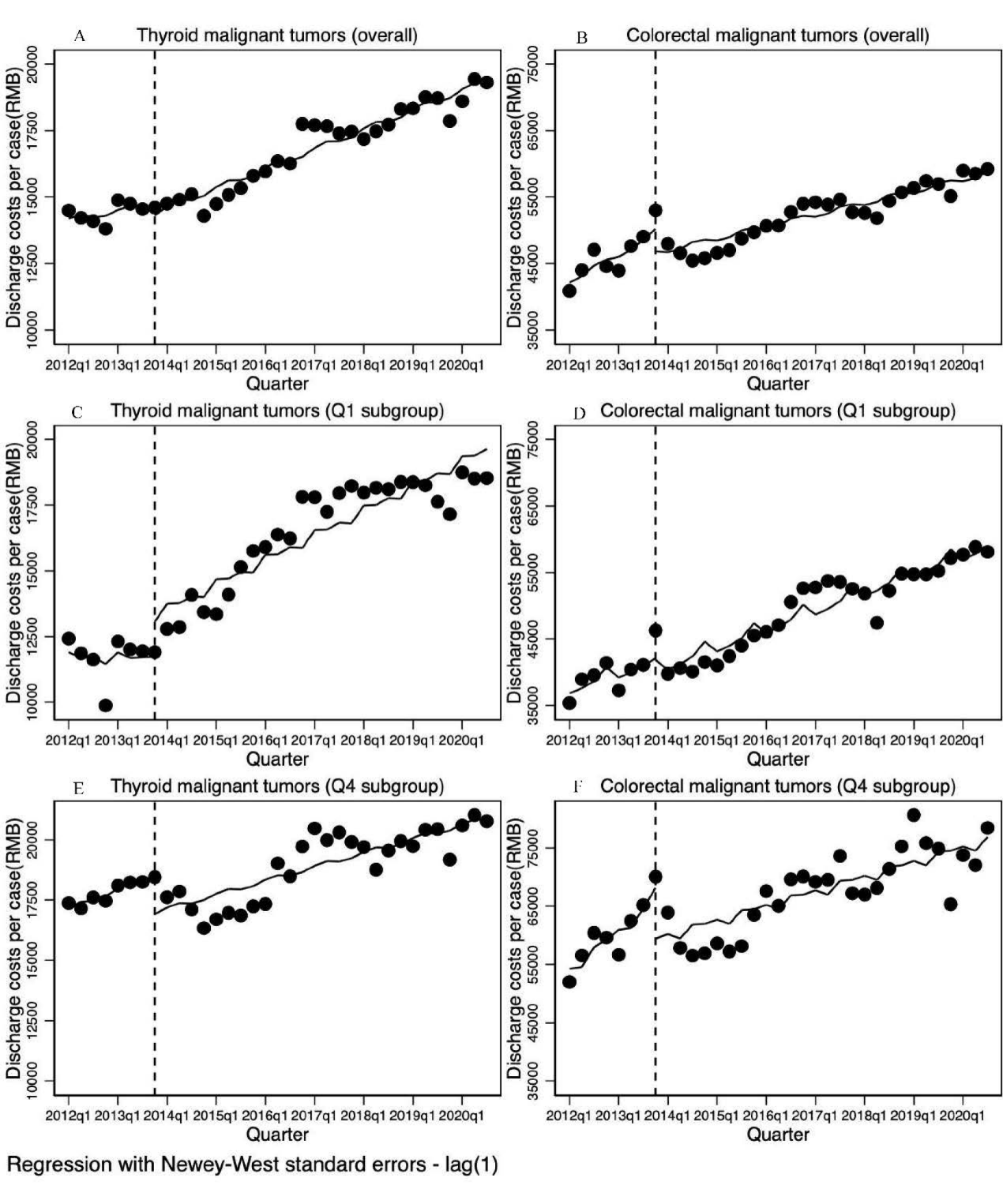


Supplementary figure 2 ITS analysis of discharge costs per case (season effects adjust)
